# Supplementary material for: Broadly neutralizing anti-HIV-1 antibodies tether viral particles at the surface of infected cells
Source: Nat Commun. 2022 Feb 2;13:630. doi: 10.1038/s41467-022-28307-7 (PMC8810770; doi:10.1038/s41467-022-28307-7)
Supplement: Supplementary file 1 — Supplementary information [file 41467_2022_28307_MOESM1_ESM.pdf]

SUPPLEMENTARY INFORMATION

Broadly neutralizing anti-HIV-1 antibodies tether viral particles at the surface of infected cells.

Jérémy Dufloo[1,2,#], Cyril Planchais[3], Stéphane Frémont[4], Valérie Lorin[3], Florence Guivel-Benhassine[1], Karl Stefic[5], Arnaud Echard[4], Nicoletta Casartelli[1], Philippe Roingeard[6], Hugo Mouquet[3], Olivier Schwartz[1,7,*] and Timothée Bruel[1,7,*]

[1] Institut Pasteur, Université de Paris, CNRS UMR3569, Virus and Immunity Unit, 75015 Paris, France

[2] Université de Paris, École doctorale BioSPC 562, 75013 Paris, France

[3] Institut Pasteur, Université de Paris, INSERM U1222, Humoral Immunology Laboratory, 75015 Paris, France

[4] Institut Pasteur, Université de Paris, CNRS UMR3691, Membrane Traffic and Cell Division Unit, 75015 Paris, France

[5] CHRU de Tours, Hôpital Bretonneau, Service de Bactériologie-Virologie, 37000 Tours, France.

[6] Université de Tours, CHRU de Tours, INSERM U1259 MAVIVH and Plateforme IBiSA de Microscopie Électronique, 37000 Tours, France

[7] Vaccine Research Institute, 94000 Créteil, France

* Corresponding authors olivier.schwartz@pasteur.fr and timothee.bruel@pasteur.fr

# Present address: Institute for Integrative Systems Biology (I2SysBio), Universitat de València-CSIC, 46980 València, Spain

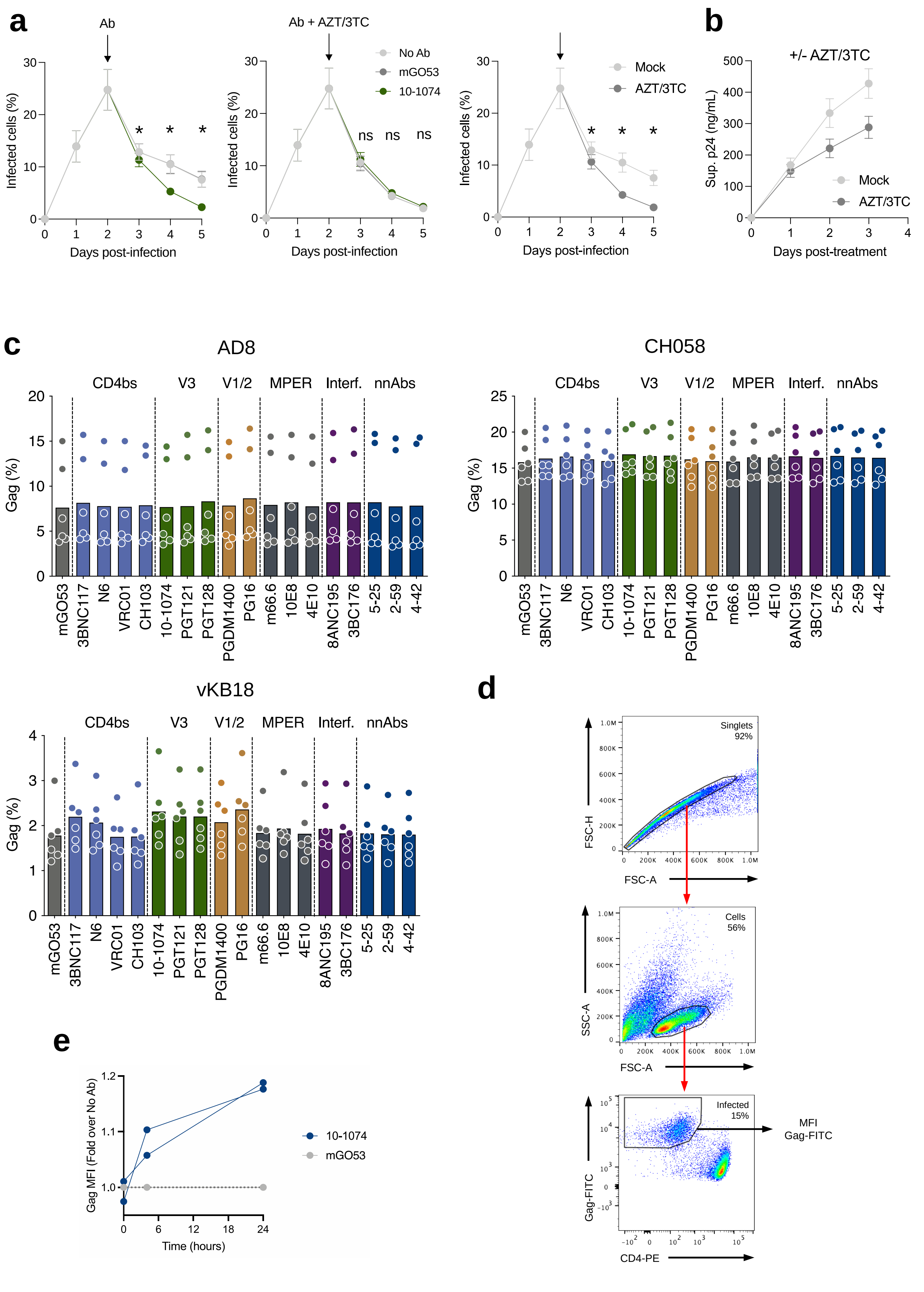

Supplementary Figure 1 (related to Figure 1). See legend next page.

Supplementary Figure 1 (related to Figure 1).

a. Primary CD4 T cells were infected with HIV-1 for 48h, washed, and cultivated for 24h with or without antibodies (no antibody [no Ab], anti-HIV-1 Env [10-1074] or isotype control [mGO53]) with (left) or without (middle) azidothymidine (AZT) and lamivudine (3TC). The right panel is a direct comparison of “no Ab” conditions with or without AZT/3TC. The frequency of Gag+ cells was measured every day by flow cytometry. Dots represent the mean of 6 donors of CD4 T cells. Error bars indicate SEM. *p=0.0313 using a Two-tailed Wilcoxon test compared to mGO53 (left panel) or no AZT/3TC (right panel).

b. Viral protein production in supernatant following treatment of infected cells with AZT/3TC. Primary CD4 T cells were infected with HIV-1 for 48h, washed, and cultivated with or without AZT/3TC. p24 was quantified by ELISA. Dots represent the mean of 6 donors of CD4 T cells. Error bars indicate SEM.

c. Primary CD4 T cells were infected with HIV-1 (strain AD8, CH058 or vKB18) and cultivated for 24h with an isotype control (mGO53) or anti-HIV-1 non-neutralizing antibodies (nnAbs) or bNAbs targeting various epitopes of the envelope, in presence of antiretrovirals (AZT and 3TC). The percentage of infected cells (CD4-Gag+) cells was measured by flow cytometry. Data represent the mean±SEM of 6 donors of CD4 T cells.

d. Flow cytometry gating strategy used to measure Gag levels in infected cells.

e. Primary CD4 T cells were infected with HIV-1 (strain AD8, CH058 or vKB18) and cultivated for 0, 6 or 24h with an isotype control (mGO53) or an anti-HIV-1 antibody (10-1074). The Mean Fluorescence Intensity (MFI) of Gag of in productively infected cells (CD4-Gag+) cells was measured by flow cytometry and normalized on the isotype control (mGO53). Data represent 2 independent donors of CD4 T cells.

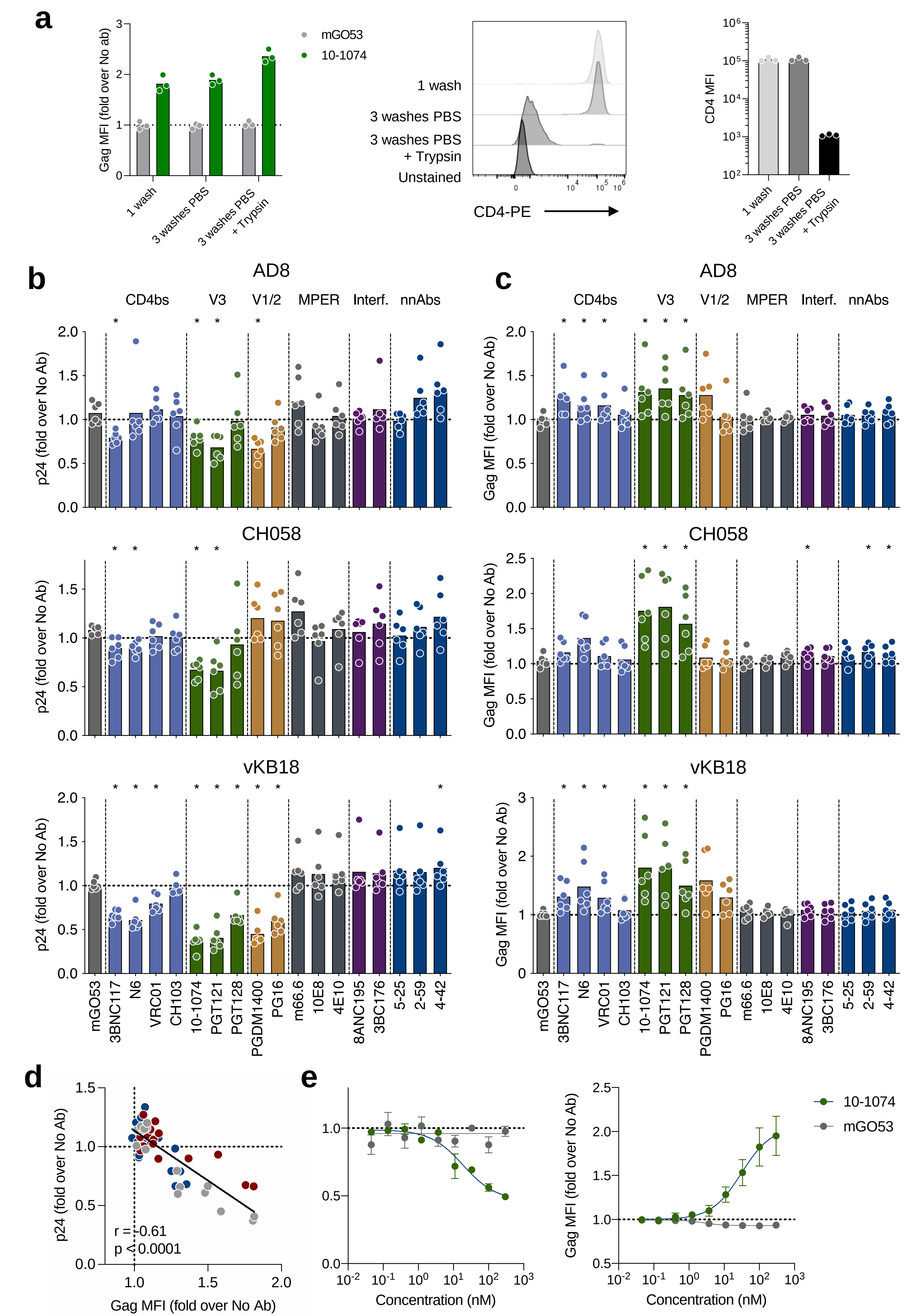

Supplementary Figure 2 (related to Figure 1). See legend next page.

Supplementary Figure 2 (related to Figure 1).
a. Cell-associated Gag Median Fluorescence Intensity (MFI) in CD4 T cells infected with CH058 and cultivated for 24h with an isotype control (mGO53) or a bNAb (10-1074). Prior to antibody treatment, infected cells were subjected to different washes: our normal procedure (1 wash), an extended procedure including 3 PBS washes, or this extended protocol followed by treatment with trypsin for 30 min at 37°C. Cell-associated Gag MFI was measured by flow cytometry and normalized to the "no antibody" condition (left panel). The efficacy of trypsin treatment was assessed by measuring surface levels of CD4 by flow cytometry (middle and right panels). Each dot represents a donor of CD4 T cells (n=3). Bars represent the mean.
b. p24 levels in the supernatants of CD4 T cells infected with different strains of HIV-1 (strain AD8, CH058 or vKB18) and cultivated for 24h with an isotype control (mGO53) or anti-HIV-1 antibodies. p24 concentrations were measured by ELISA and normalized to the "no antibody" condition. Each dot represents a donor of CD4 T cells (n=6). Bars represent the mean. *p=0.0313 (Two-tailed Wilcoxon test compared to mGO53).
c. Cell-associated Gag Median Fluorescence Intensity (MFI) in CD4 T cells infected with different strains of HIV-1 (AD8, CH058 or vKB18) and cultivated for 24h with an isotype control (mGO53) or anti-HIV-1 antibodies. Cell-associated Gag MFI was measured by flow cytometry and normalized to the "no antibody" condition. Each dot represents a donor of CD4 T cells (n=6). Bars represent the mean. *p=0.0313 (Two-tailed Wilcoxon test compared to mGO53).
d. Correlation between the fold change in cell-associated Gag and p24 release by infected cells. Each dot represents a different antibody (n=18). Mean values of 6 donors of CD4 T cells are depicted. Data from cells infected with 3 different viral strains are represented with different colors (blue: AD8; red: CH058; grey: vKB18). A two-tailed Spearman correlation test was performed, and the correlation r and p-value are indicated.
e. p24 levels in supernatants (right) and cell-associated Gag MFI (left) of HIV-1-infected CD4 T cells (CH058) cultivated for 24h with the indicated concentration of an isotype control (mGO53) or a bNAb (10-1074). p24 concentrations were measured by ELISA and cell-associated Gag MFI was measured by flow cytometry. All values were normalized to the "no antibody" condition. Data represent the mean±SEM of n=5 (left panel) or n=4 (right panel) donors of CD4 T cells.

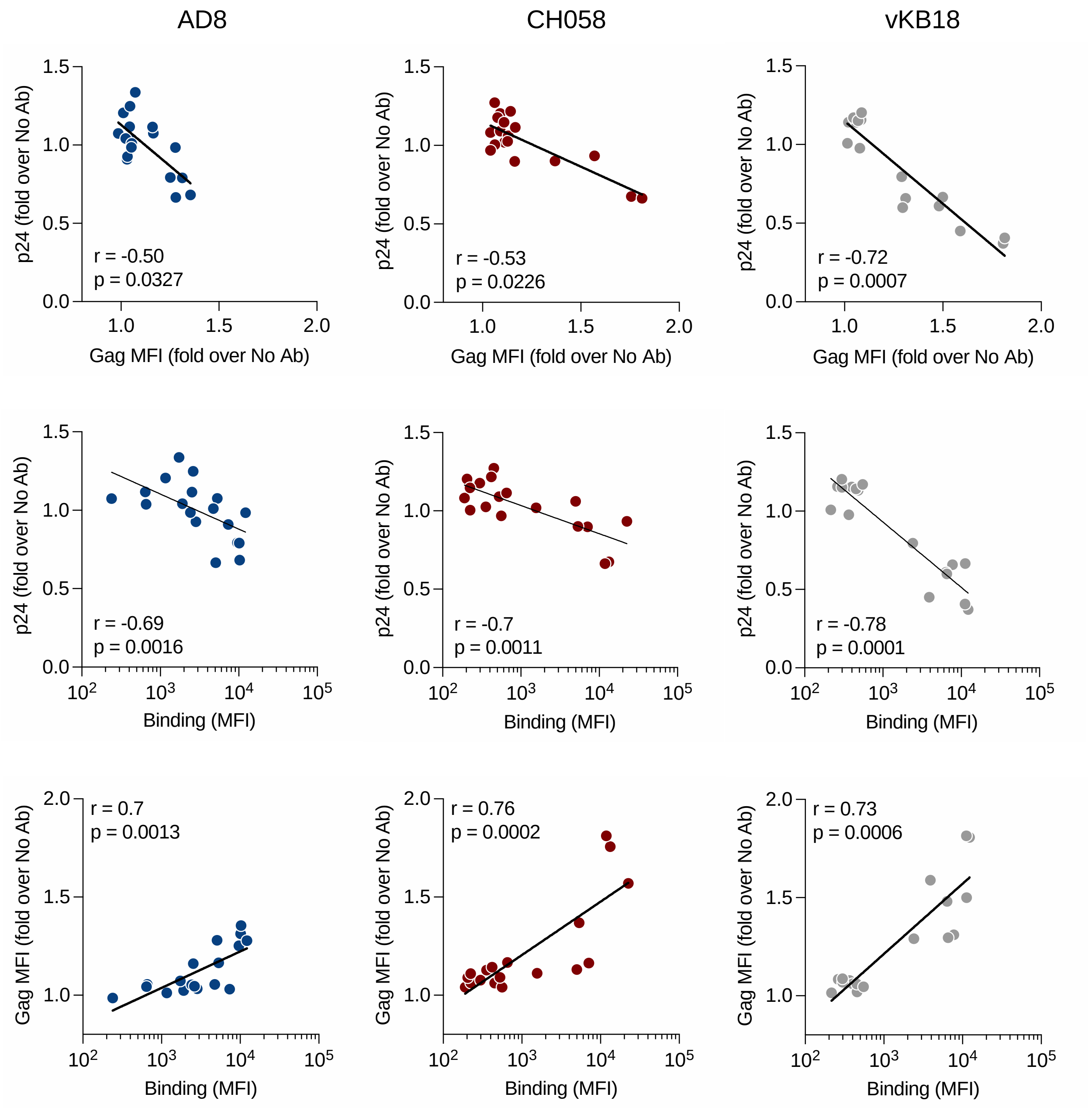

Supplementary Figure 3 (related to Figure 1).

Correlation between the fold change in cell-associated Gag, the fold change in supernatant p24 levels and antibody binding to infected cells. Data from cells infected with HIV-1 strains AD8 (blue, left panels), CH058 (red, middle panels) and vKB18 (grey, right panels) are represented. Each dot represents a different antibody (n=18). Mean values of 6 donors of CD4 T cells are depicted. A two-tailed Spearman correlation test was performed, and the correlation r and p-value are indicated.

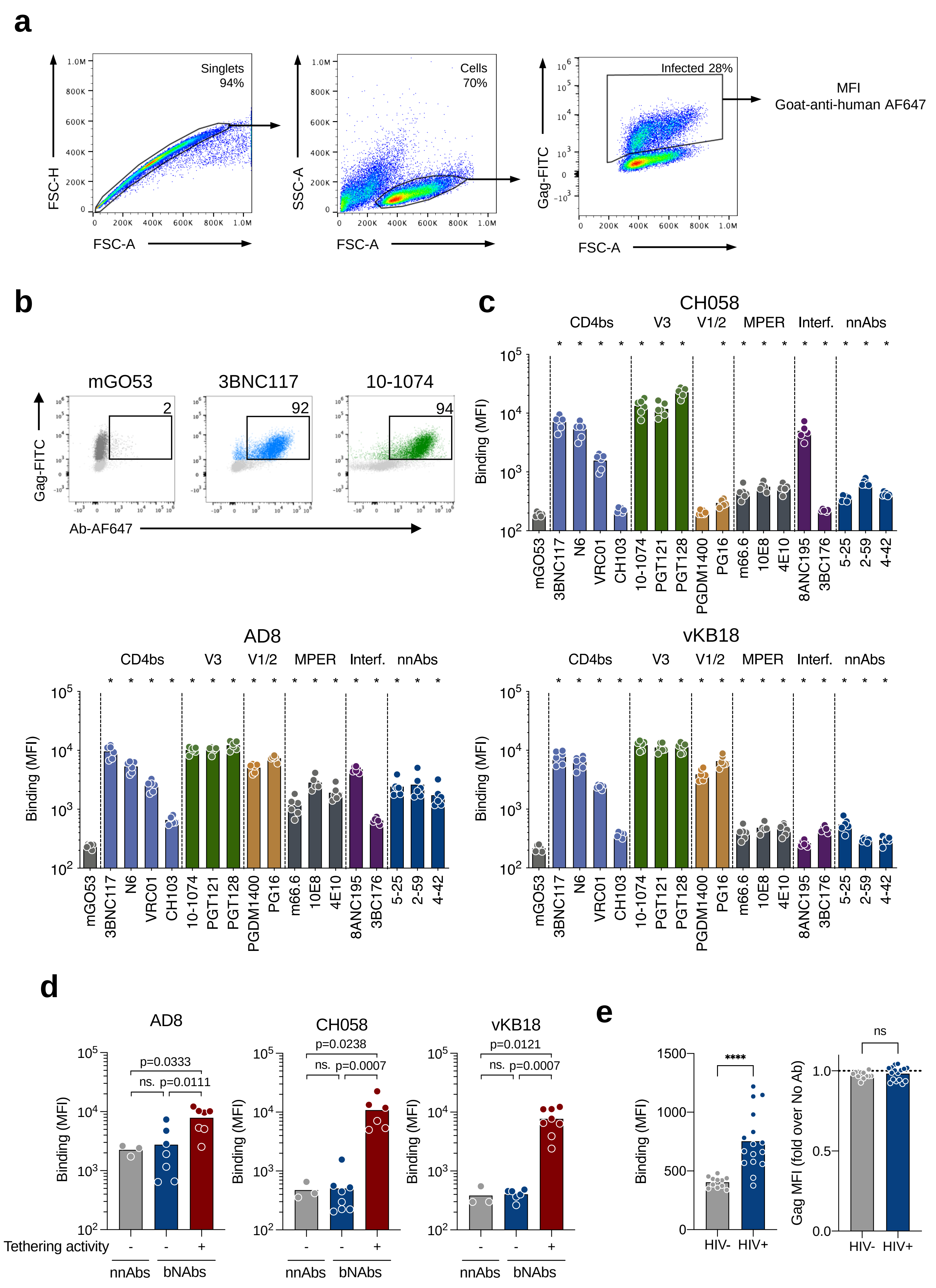

Supplementary Figure 4 (related to Figure 1). See legend next page.

Supplementary Figure 4 (related to Figure 1).
a. Flow cytometry gating strategy to measure antibody binding to infected cells
b. Representative flow cytometry analysis of antibody binding to infected (CH058) CD4 T cells. Percentages of antibody-positive Gag$^+$ cells are indicated.
c. Primary CD4 T cells were infected with HIV-1 (strain AD8, CH058 or vKB18). The Median Fluorescence Intensity (MFI) of binding of an isotype control (mGO53) or anti-HIV-1 non-neutralizing antibodies (nnAbs) or bNAbs targeting various epitopes of the envelope (CD4 binding site [CD4bs], glycans-V3 loop, glycans-V1/V2 loop, Membrane Proximal External Region [MPER], gp120/gp41 interface) was measured by flow cytometry. Each dot represents a donor of CD4 T cells (n=6). Bars represent the mean. *p=0.0313 (Two-tailed Wilcoxon test compared to mGO53).
d. The MFI of binding to infected cells of bNAbs and nnAbs was compared, according to their capacity to inhibit viral release. Each dot represents an antibody (mean of n=6 donors of CD4 T cells). p-values are indicated on the figure (Two-tailed Mann-Whitney test).
e. HIV-1-infected (CH058) CD4 T cells were cultivated with healthy (HIV-; n=11) or HIV-1-infected ART-treated (HIV+; n=16) patients' sera (diluted 1:100). Antibody binding (left) and fold change in cell-associated Gag (middle) were measured. Each dot is the mean of 3 donors of CD4 T cells. ****p<0.0001; ns not significant (Two-tailed Mann-Whitney test).

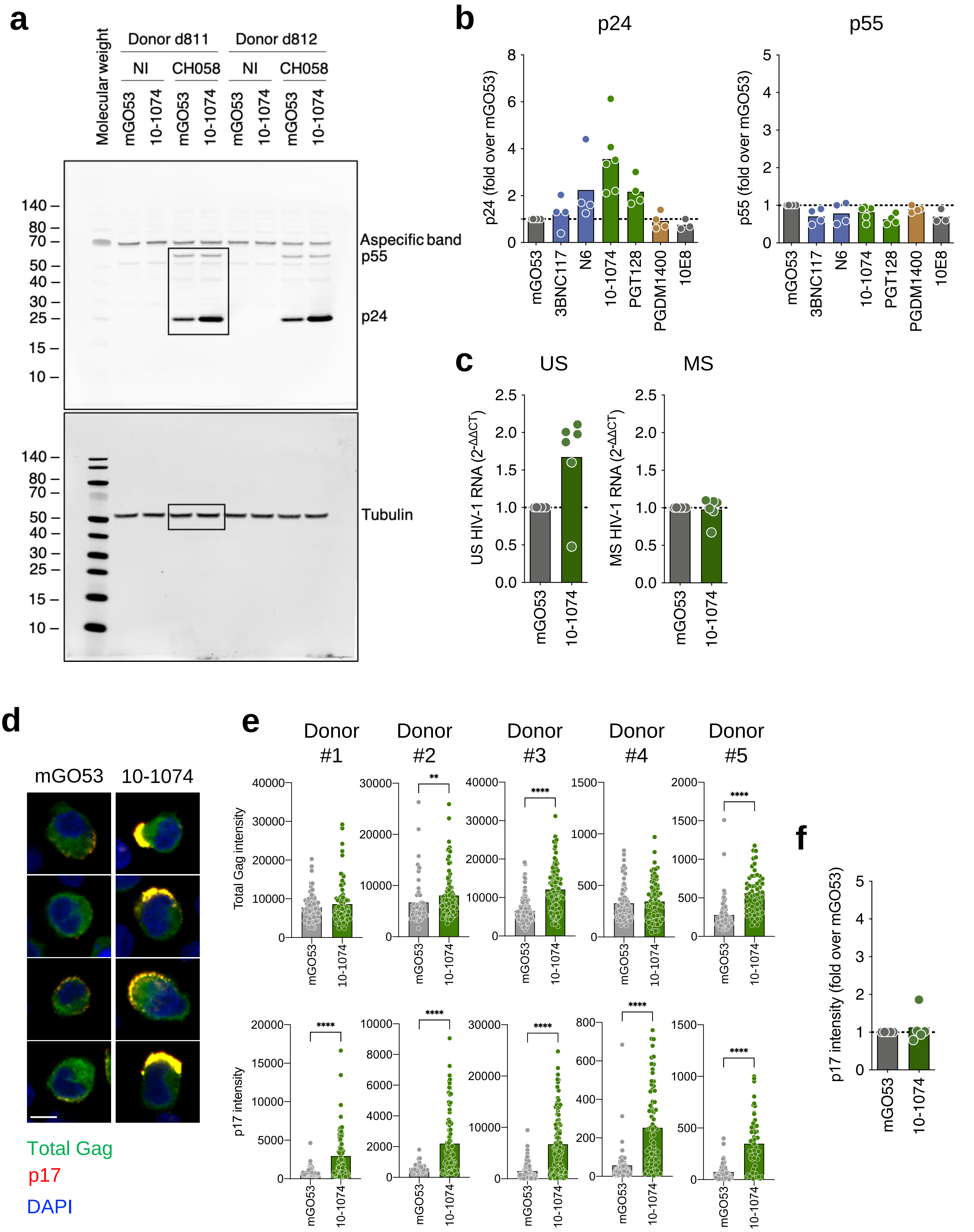

Supplementary Figure 5 (related to Figure 2). See legend next page.

Supplementary Figure 5 (related to Figure 2).

a. Unprocessed western blot shown in Figure 2A (the black squares show the cropped regions). Data from 2 donors of CD4 T cells infected (CH058) or not (NI) and cultivated with an isotype control (mGO53) or a bNAb (10-1074) for 24h are shown. The molecular weights (kDa) are shown. One representative experiment (out of three) is shown.

b. Western blot analysis of p24 and p55 levels in HIV-1-infected CD4 T cells (CH058) cultivated for 24h with an isotype control (mGO53) or anti-HIV-1 bNAbs. The color of the bar indicates the Env epitope targeted by the bNAb (blue: CD4bs; green: V3 loop; yellow: V1/V2 loop; grey: MPER). Each dot represents a donor of CD4 T cells (n=3 for 10E8; n=4 for 3BNC117, N6, PGT128 and PGDM1400; n=6 for mGO53 and 10-1074). Bars represent the mean.

c. Quantitative real-time PCR quantification of unspliced (US; left) and multiply spliced (MS; right) HIV-1 RNA. Data were normalized to a housekeeping gene (GAPDH) and to the isotype control condition (mGO53) using the $2^{-\Delta\Delta CT}$ method. Each dot represents a donor of CD4 T cells (n=6). Bars represent the mean.

d. Representative confocal microscopy images of infected CD4 T cells (CH058) cultivated for 24h with an isotype control (mGO53) or with a bNAb (10-1074). Cells were stained for intracellular total Gag (green) and p17 (red). Nuclei were stained with DAPI (blue). Scale bar: 5μm. One representative experiment (out of five) is shown.

e. Confocal microscopy analysis of total Gag (top) and p17 (bottom) levels in HIV-1-infected CD4 T cells (CH058) cultivated for 24 hours with an isotype control (mGO53) or a bNAb (10-1074). Each dot represents an individual cell (Donor #1: n=67 (mGO53) and n=76 (10-1074); Donor #2: n=49 (mGO53) and n=93 (10-1074); Donor #3: n=138 (mGO53) and n=102 (10-1074); Donor #4: n=76 (mGO53) and n=75 (10-1074); Donor #5: n=70 (mGO53) and n=57 (10-1074)). Analysis in 5 donors of CD4 T cells is shown. **p=0.0037; ****p<0.0001 (Two-tailed Mann-Whitney test).

f. Confocal microscopy analysis of p17 levels in non-infected bystander cells (in a culture of CH058-infected cells) cultivated for 24 hours with an isotype control (mGO53) or a bNAb (10-1074). Each dot represents a donor of CD4 T cells (n=5). 30 cells were analyzed per donor.

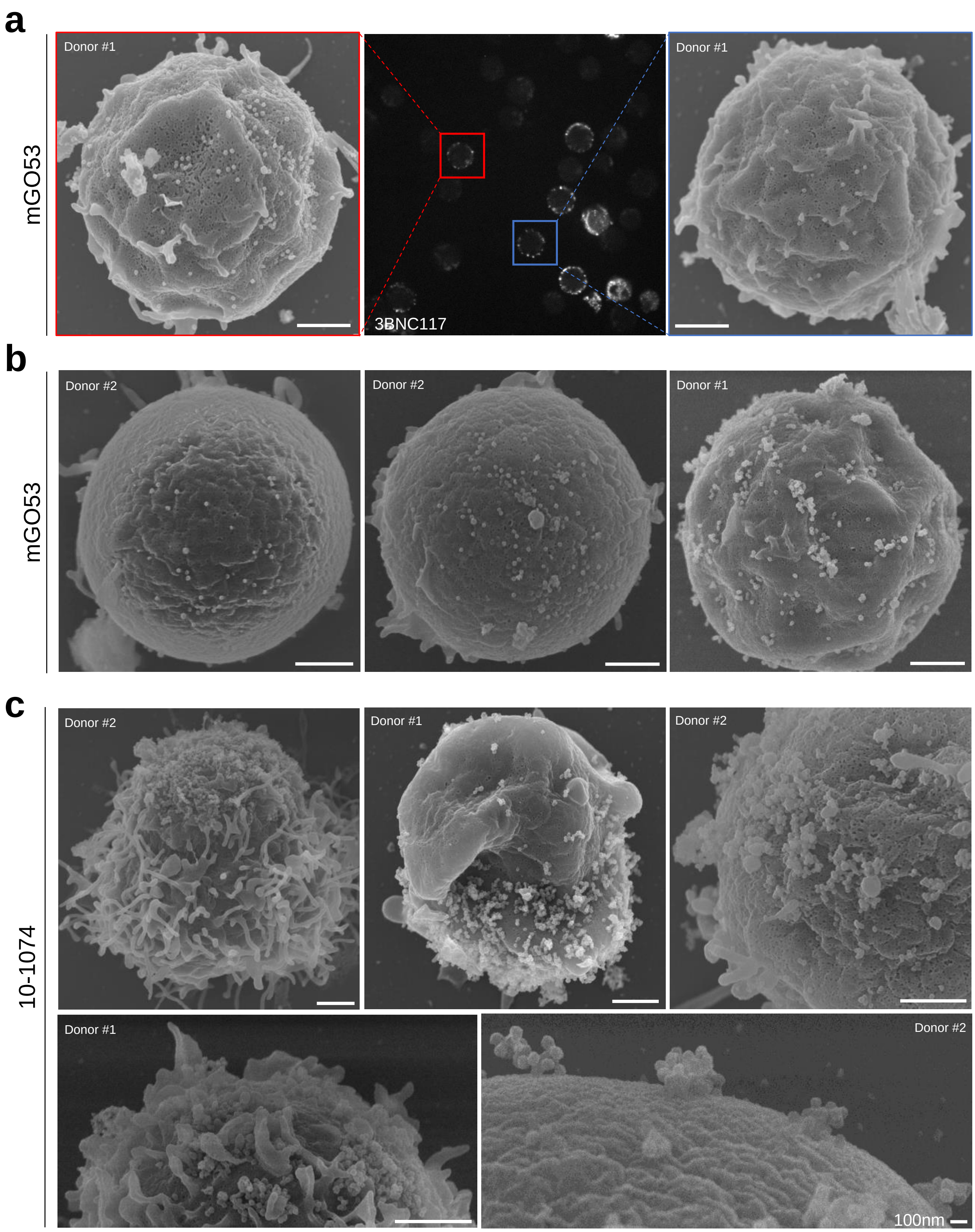

Supplementary Figure 6 (related to Figure 2).

a. HIV-1-infected CD4 T cells (CH058) were identified and located by confocal microscopy with an envelope staining using an anti-HIV-1 Env antibody (3BNC117). The same cells were imaged using scanning electron microscopy (SEM). Examples are shown with two cells from the same donor cultivated with an isotype control (mGO53). Scale bar: 1µm.

b. Representative SEM images of HIV-1-infected CD4 T cells (CH058) cultivated for 24h with an isotype control (mGO53). Scale bar: 1µm.

c. Representative SEM images of HIV-1-infected CD4 T cells (CH058) cultivated for 24h with a bNAb (10-1074). Scale bar: 1µm, unless otherwise stated. The CD4 T cell donor is indicated for each image.

Representative images from two independent donors are shown.

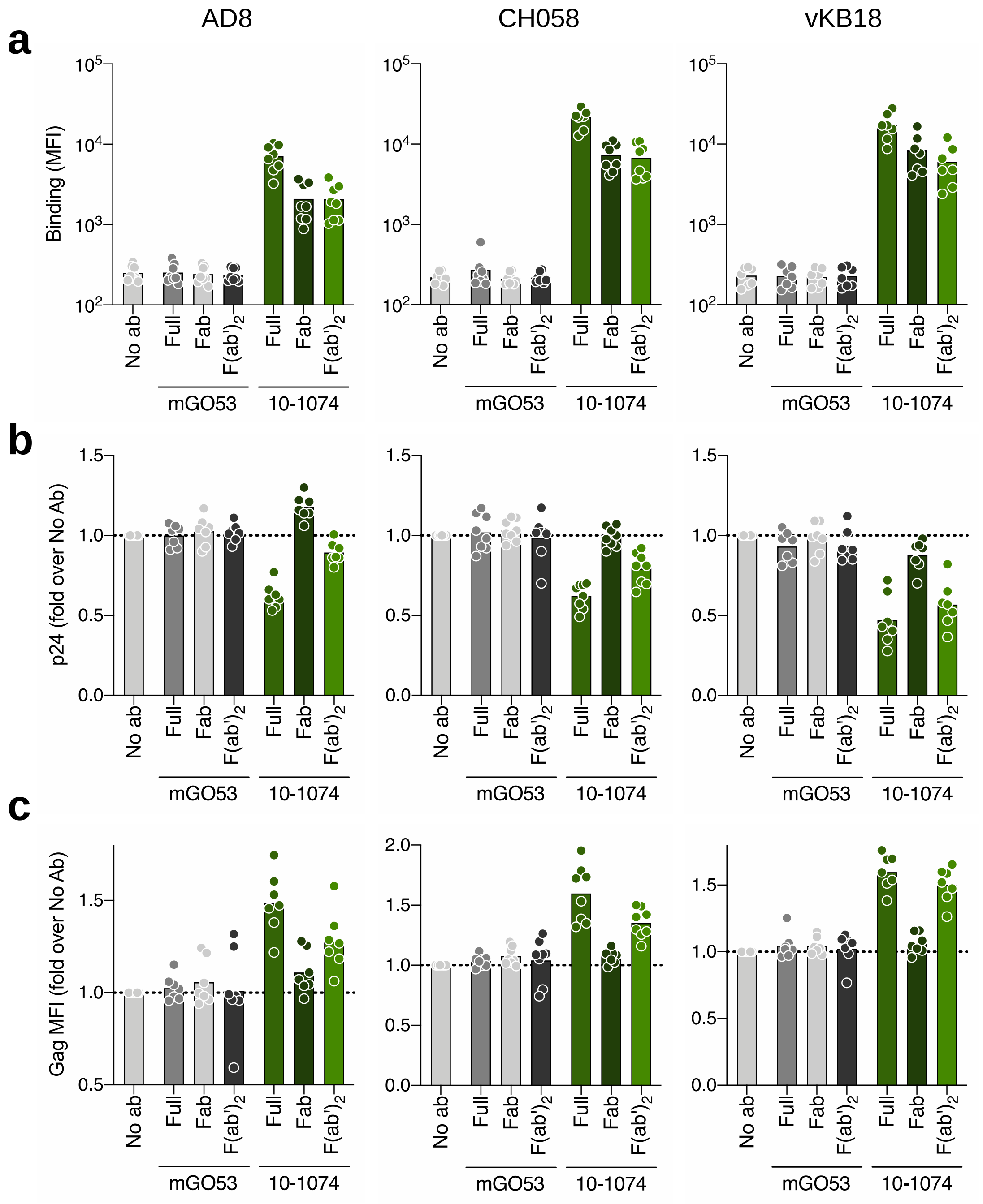

Supplementary Figure 7 (related to Figure 3).

CD4 T cells were infected with various strains of HIV-1 (strains AD8, CH058 or vKB18).

a. Binding Median Fluorescence Intensity (MFI) of Full, Fab or $(Fab')_2$ fragments of an isotype control (mGO53) or a bNAb (10-1074) was measured by flow cytometry. A "no antibody" condition (No ab) was used as a control. Each dot represents a donor of CD4 T cells (n=7 for vKB18 and n=8 for AD8 and CH058).

b. p24 levels in the supernatants of infected CD4 T cells cultivated for 24h with Full, Fab or $(Fab')_2$ fragments of an isotype control (mGO53) or a bNAb (10-1074). p24 concentrations were measured by ELISA and normalized to the "no antibody" condition. Each dot represents a donor of CD4 T cells (n=7 for AD8 and vKB18 and n=8 for CH058).

c. Cell-associated Gag MFI in infected CD4 T cells cultivated for 24h with Full, Fab or $(Fab')_2$ fragments of an isotype control (mGO53) or a bNAb (10-1074). Gag MFI was measured by flow cytometry and normalized to the "no antibody" condition. Each dot represents a donor of CD4 T cells (n=7 for AD8 and vKB18 and n=8 for CH058).

All antibodies were used at a concentration of 100 nM.

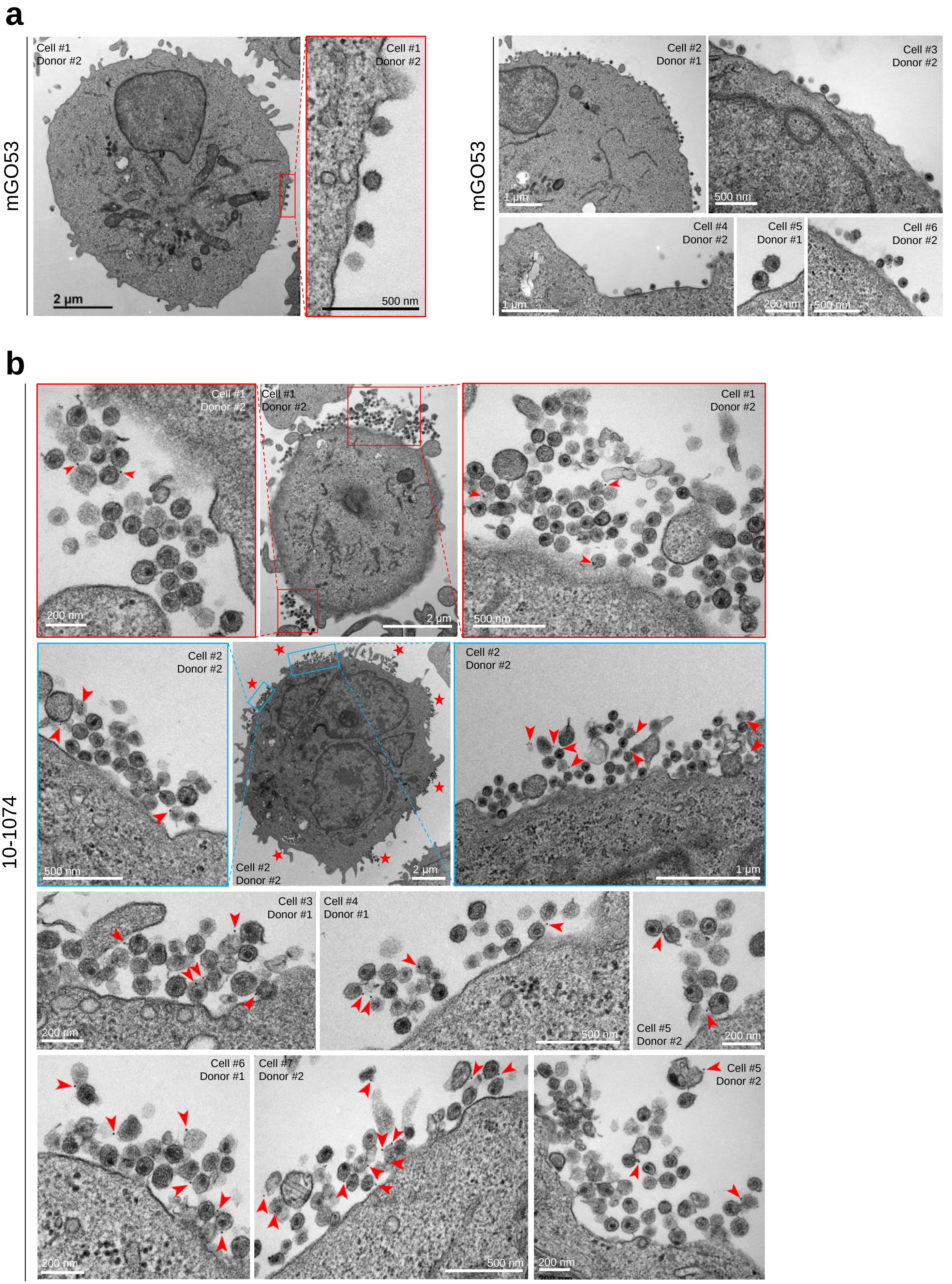

Supplementary Figure 8 (related to Figure 3). See legend next page.

Supplementary Figure 8 (related to Figure 3).
Infected CD4 T cells (CH058) cultivated for 24h with an isotype control (mGO53) or with a bNAb (10-1074) were stained with an anti-human IgG antibody coupled to colloidal gold beads and analyzed by transmission electron microscopy (TEM).
a. Representative TEM images of HIV-1-infected cells (CH058) cultivated for 24h with an isotype control (mGO53). A magnification of membrane-associated viral particles is shown.
b. Representative TEM images of HIV-1-infected cells (CH058) cultivated for 24h with a bNAb (10-1074). Magnifications of areas with viral accumulation are shown.
Scale bar: 200nm-2µm. The cell donor and the cell ID is indicated for each image. Red stars indicate areas of viral retention. Red arrowheads point colloidal gold beads, indicative of 10-1074.
Representative images from two independent donors are shown.

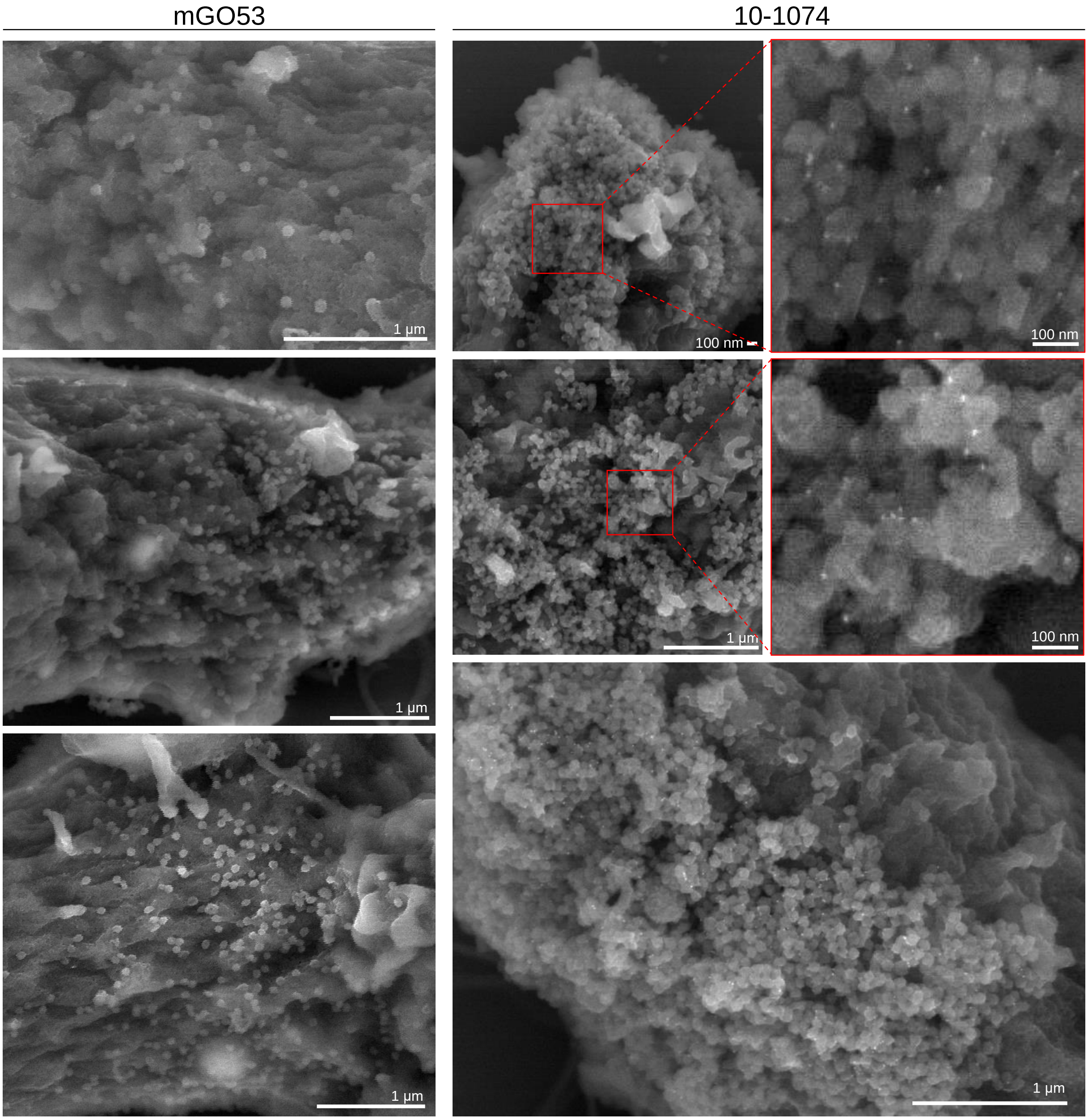

Supplementary Figure 9 (related to Figure 3).

Infected CD4 T cells (CH058) cultivated for 24h with an isotype control (mGO53) or with a bNAb (10-1074) were stained with an anti-human IgG antibody coupled to colloidal gold and analyzed by scanning electron microscopy. Representative images are shown. Bright white dots are the colloidal gold beads, indicative of 10-1074. Magnifications on areas with strong colloidal gold staining are shown. Scale bars: 100nm or 1µm. A representative experiment (out of two) is shown.

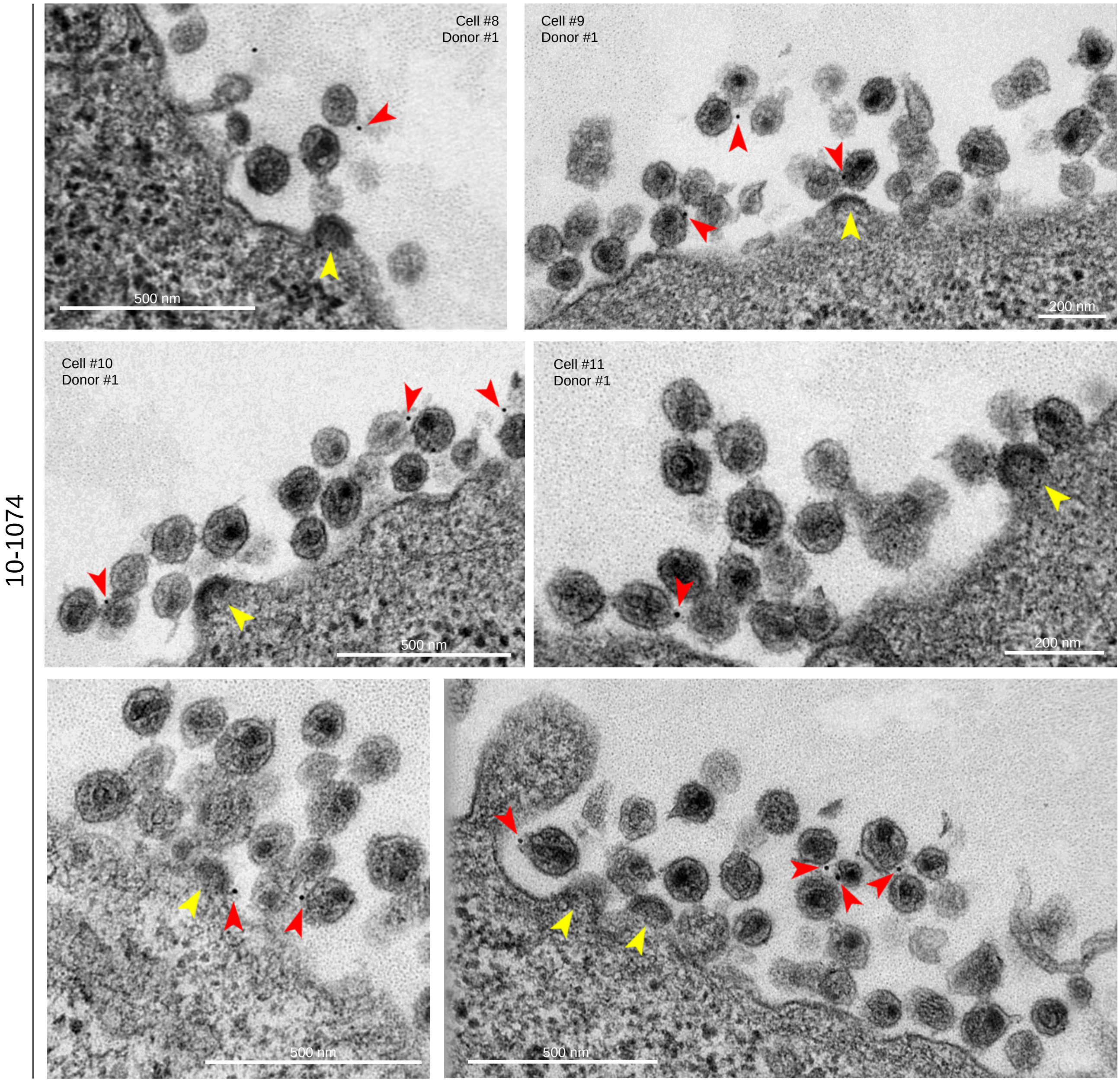

Supplementary Figure 10 (related to Figure 3).
Infected CD4 T cells (CH058) cultivated for 24h with a bNAb (10-1074) were stained with an anti-human IgG antibody coupled to colloidal gold beads and analyzed by transmission electron microscopy (TEM). Magnifications of areas with viral accumulation at budding sites are shown.
Scale bar: 200nm-500nm. The cell donor and the cell ID are indicated for each image. Red arrowheads point colloidal gold beads, indicative of 10-1074. Yellow arrowheads indicate budding virions. A representative experiment (out of two) is shown.

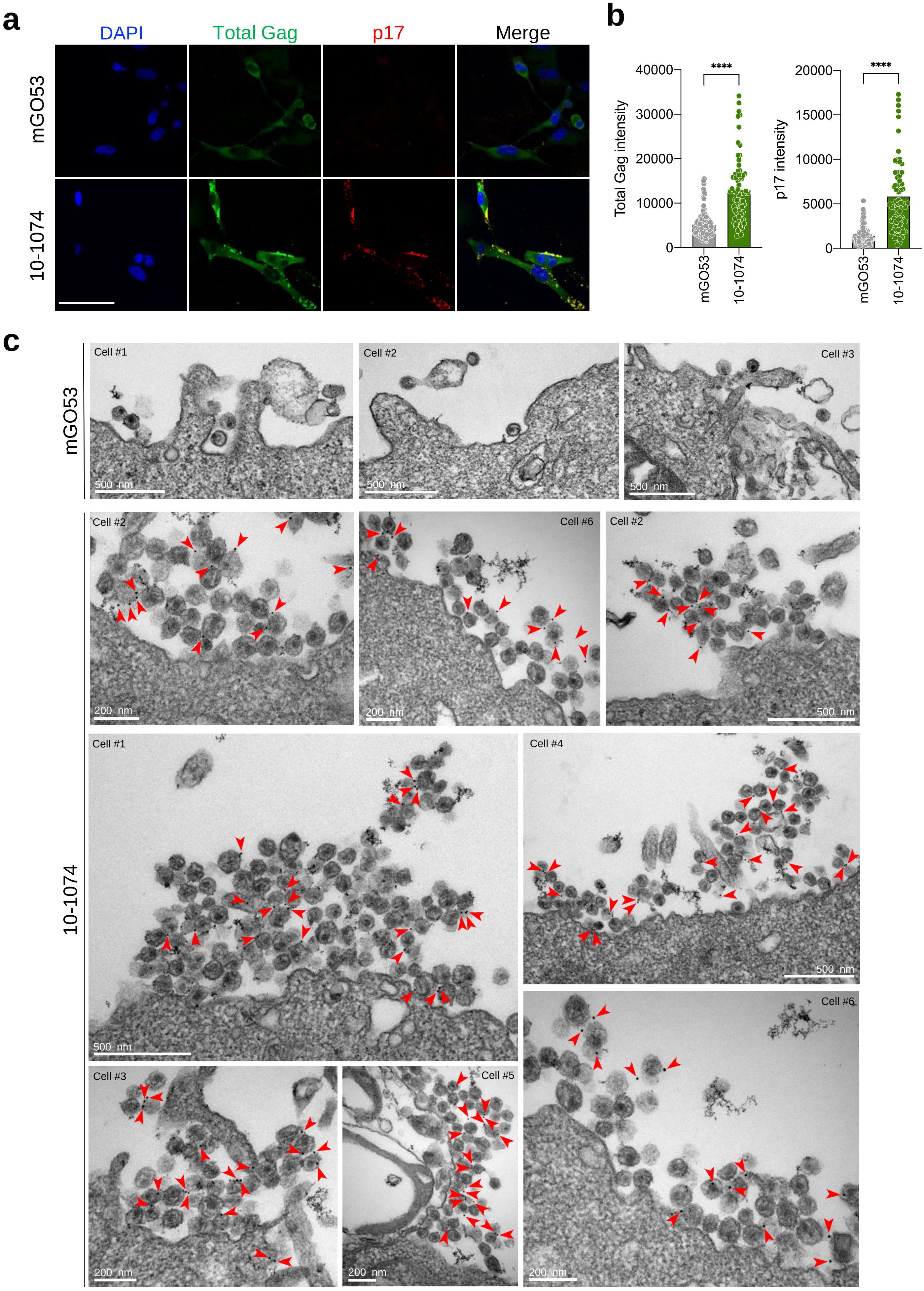

Supplementary Figure 11 (related to Figure 3). See legend next page.

Supplementary Figure 11 (related to Figure 3).
a. Representative confocal microscopy images of infected (CH058) CHME cells cultivated for 24h with an isotype control (mGO53) or with a bNAb (10-1074). Cells were stained for intracellular total Gag (green) and p17 (red). Nuclei were stained with DAPI (blue). Scale bar: 100µm. A representative experiment (out of two) is shown.
b. Confocal microscopy analysis of total Gag (left) and p17 (right) levels in HIV-1-infected CHME cells (CH058) cultivated for 24 hours with an isotype control (mGO53) or a bNAb (10-1074). Each dot represents an individual cell (n=121 in mGO53 and n=71 in 10-1074). ****$p<0.0001$ (Two-tailed Mann-Whitney test).
c. Infected CHME cells (CH058) cultivated for 24h with an isotype control (mGO53) or with a bNAb (10-1074) were stained with an anti-human IgG antibody coupled to colloidal gold beads, and analyzed by transmission electron microscopy. Representative images are shown. Red arrowheads point colloidal gold beads, indicative of 10-1074.

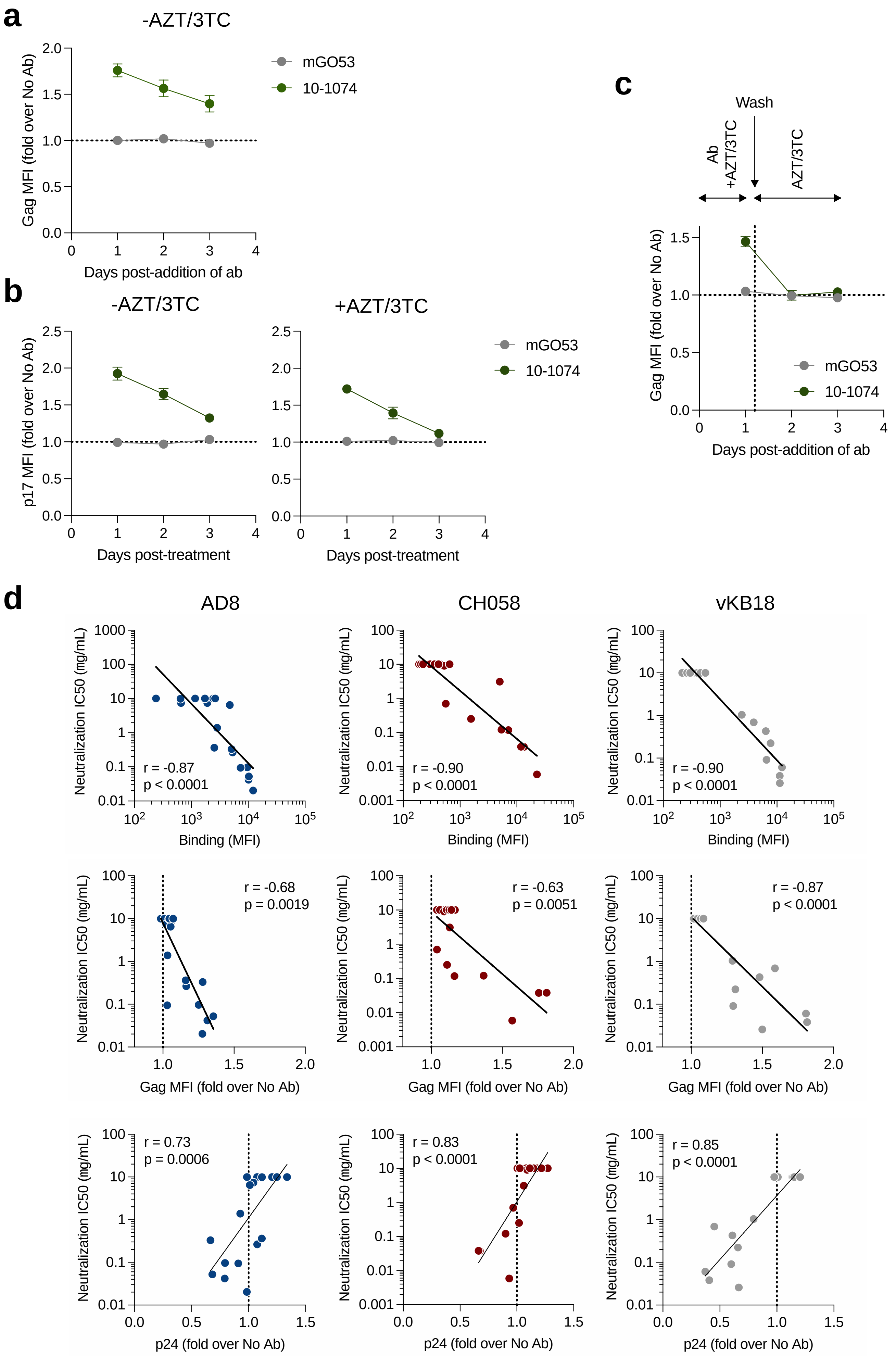

Supplementary Figure 12 (related to Figure 4). See legend next page.

Supplementary Figure 12 (related to Figure 4).

a. Primary CD4 T cells were infected with HIV-1 for 48h and were then cultivated with 10-1074 without AZT/3TC. Fold-change in cell-associated Gag (right) was measured after 1, 2 and 3 days of culture by flow cytometry. The MFI of Gag staining was normalized to the “no antibody” condition. Dots represent the mean±SEM of 6 donors of CD4 T cells.

b. Primary CD4 T cells were infected with HIV-1 for 48h and were then cultivated with 10-1074 without (left) or with (right) AZT/3TC. Fold-change in cell-associated p17 (right) were measured after 1, 2 and 3 days of culture by flow cytometry and normalized to the “no antibody” condition. Dots represent the mean±SEM of 3 donors of CD4 T cells.

c. Primary CD4 T cells were infected with HIV-1 for 48h and then cultivated with 10-1074 in the presence of AZT/3TC for 24h to form aggregates. Cells were then washed to remove 10-1074 and the stability of aggregates was followed by measuring Gag levels over 2 days. The MFI of Gag staining was measured by flow cytometry and normalized to the “no antibody” condition. Dots represent the mean±SEM of 3 donors of CD4 T cells.

d. Correlation between the fold change in cell-associated Gag, the fold change in supernatant p24 levels and antibody binding to infected cells. Data from cells infected with HIV-1 strains AD8 (blue, left panels), CH058 (red, middle panels) and vKB18 (grey, right panels) are represented. Each dot represents a different antibody (n=18). Mean values of 6 donors of CD4 T cells are depicted. Two-tailed Spearman correlation tests were performed, and the correlation r and p-value are indicated.

Supplementary Table 1. Anti-HIV-1 monoclonal antibodies used in this study.

Antibody	bNAb/nnAb	Epitope	Reference
mGO53	Isotype control		[56]
3BNC117	bNAb	CD4 binding site	[57]
N6	bNAb	CD4 binding site	[58]
VRC01	bNAb	CD4 binding site	[59]
CH103	bNAb	CD4 binding site	[60]
10-1074	bNAb	V3 loop	[61]
PGT121	bNAb	V3 loop	[62]
PGT128	bNAb	V3 loop	[62]
PGDM1400	bNAb	V1/V2 loops	[63]
PG16	bNAb	V1/V2 loops	[64]
m66.6	bNAb	MPER (gp41)	[65]
10E8	bNAb	MPER (gp41)	[66]
4E10	bNAb	MPER (gp41)	[67]
8ANC195	bNAb	gp120/41 interface	[57]
3BC176	bNAb	gp120/41 interface	[68]
5-25	nnAb	Gp41 immunodominant	[69]
2-59	nnAb	V3 crown	[69]
4-42	nnAb	Co-receptor binding site	[69]

Supplementary Table 2. Characteristics of HIV-1-infected individuals

Patient ID	Age	Gender	Time on ART (years)	RNA viral load (copies/mL)	CD4 T cell count (cells/mm^3)
KB24	NA	NA	NA	NA	NA
KB25	NA	NA	NA	NA	NA
KB26	NA	NA	NA	NA	NA
KB30	39	Male	5	<40	730
KB31	60	Male	8	<40	484
KB32	57	Male	22	<40	1024
KB33	63	Male	8	<40	959
KB34	49	Female	9	<40	505
KB36	66	Male	7	<40	672
KB37	49	Male	19	<40	1025
KB38	50	Female	7	<40	640
KB40	57	Female	8	<40	563
KB42	39	Male	3	<40	1329
KB43	55	Female	17	<40	602
KB44	56	Female	7	<40	806
KB45	53	Female	10	<40	1033

NA: not available; ART: antiretroviral therapy
