# Supplementary material for: Broadly neutralizing anti-HIV-1 antibodies tether viral particles at the surface of infected cells
Source: Nat Commun. 2022 Feb 2;13:630. doi: 10.1038/s41467-022-28307-7 (PMC8810770; doi:10.1038/s41467-022-28307-7)
Supplement: Supplementary file 3 — Reporting Summary [file 41467_2022_28307_MOESM3_ESM.pdf]

nature portfolio

Corresponding author(s): Timothée Bruel and Olivier Schwartz

Last updated by author(s): Jan 11, 2022

Reporting Summary

Nature Portfolio wishes to improve the reproducibility of the work that we publish. This form provides structure for consistency and transparency in reporting. For further information on Nature Portfolio policies, see our Editorial Policies and the Editorial Policy Checklist.

Statistics

For all statistical analyses, confirm that the following items are present in the figure legend, table legend, main text, or Methods section.

n/a	Confirmed	
☐	☒	The exact sample size (*n*) for each experimental group/condition, given as a discrete number and unit of measurement
☐	☒	A statement on whether measurements were taken from distinct samples or whether the same sample was measured repeatedly
☐	☒	The statistical test(s) used AND whether they are one- or two-sided<br>*Only common tests should be described solely by name; describe more complex techniques in the Methods section.*
☒	☐	A description of all covariates tested
☒	☐	A description of any assumptions or corrections, such as tests of normality and adjustment for multiple comparisons
☐	☒	A full description of the statistical parameters including central tendency (e.g. means) or other basic estimates (e.g. regression coefficient) AND variation (e.g. standard deviation) or associated estimates of uncertainty (e.g. confidence intervals)
☐	☒	For null hypothesis testing, the test statistic (e.g. *F*, *t*, *r*) with confidence intervals, effect sizes, degrees of freedom and *P* value noted<br>*Give P values as exact values whenever suitable.*
☒	☐	For Bayesian analysis, information on the choice of priors and Markov chain Monte Carlo settings
☒	☐	For hierarchical and complex designs, identification of the appropriate level for tests and full reporting of outcomes
☒	☐	Estimates of effect sizes (e.g. Cohen's *d*, Pearson's *r*), indicating how they were calculated

Our web collection on statistics for biologists contains articles on many of the points above.

Software and code

Policy information about availability of computer code

Data collection	Attune Nxt Software v3.2.1 (ThermoFisher)<br>ZEN Software v2.0 (ZEISS)
Data analysis	Fiji v2.1.1<br>ImageStudioLite v5.2.5 (LI-COR)<br>Excel 365 v16.46 (Microsoft)<br>Prism v9.0.2 (GraphPad Software)<br>Flowjo Software v10.7.1

For manuscripts utilizing custom algorithms or software that are central to the research but not yet described in published literature, software must be made available to editors and reviewers. We strongly encourage code deposition in a community repository (e.g. GitHub). See the Nature Portfolio guidelines for submitting code & software for further information.

Data

Policy information about availability of data

All manuscripts must include a data availability statement. This statement should provide the following information, where applicable:

- Accession codes, unique identifiers, or web links for publicly available datasets
- A description of any restrictions on data availability
- For clinical datasets or third party data, please ensure that the statement adheres to our policy

All data supporting the findings of this study are available within the Article. Source data are provided with this paper.

Field-specific reporting

Please select the one below that is the best fit for your research. If you are not sure, read the appropriate sections before making your selection.

- [x] Life sciences
- [ ] Behavioural & social sciences
- [ ] Ecological, evolutionary & environmental sciences

For a reference copy of the document with all sections, see nature.com/documents/nr-reporting-summary-flat.pdf

Life sciences study design

All studies must disclose on these points even when the disclosure is negative.

Sample size	In vitro data were usually obtained from at least 6 primary cell donors, unless otherwise stated. No statistical method was used to predetermine sample size. All available samples were used in the study. Such sample sizes are typical for in vitro experiments and sufficient for a robust statistical analysis.
Data exclusions	No data were excluded.
Replication	All experiments were performed and verified multiple times or with several donors as indicated in the figure legends.
Randomization	Randomization was not relevant for this study as, for each biological replicate, antibody-treated or untreated infected cells were derived from the same donor.
Blinding	Experiment were performed without blinding due to personnel limitation. Analyses were objective and did not require blinding.

Reporting for specific materials, systems and methods

We require information from authors about some types of materials, experimental systems and methods used in many studies. Here, indicate whether each material, system or method listed is relevant to your study. If you are not sure if a list item applies to your research, read the appropriate section before selecting a response.

Materials & experimental systems

n/a	Involved in the study	
☐	☒	Antibodies
☐	☒	Eukaryotic cell lines
☒	☐	Palaeontology and archaeology
☒	☐	Animals and other organisms
☐	☒	Human research participants
☒	☐	Clinical data
☒	☐	Dual use research of concern

Methods

n/a	Involved in the study	
☒	☐	ChIP-seq
☐	☒	Flow cytometry
☒	☐	MRI-based neuroimaging

Antibodies

Antibodies used

Anti-HIV-1 antibodies (3BNC117, N6, VRC01, CH103, 10-1074, PGT121, PGT128, PGDM1400, PG16, m66.6, 10E8, 4E10, 8ANC195, 3BC176, 5-25, 2-59, 4-42 and isotype control mGO53) are human anti-Env monoclonal antibodies produced by Hugo Mouquet (Institut Pasteur).
PE-conjugated anti-CD4 antibody (clone VIT4; Miltenyi)
FITC-conjugated anti-Gag antibody (clone KC57; Beckman-Coulter)
Anti-alpha-Tubulin (clone AA13; Sigma-Aldrich)
Polyclonal anti-p24 (NIH AIDS Reagent Program; Cat# ARP-4250)
DyLight 680-conjugated Goat anti-Mouse IgG (Invitrogen; Cat#35518)
DyLight 800-conjugated Goat anti-Rabbit IgG (Invitrogen; Cat#SA5-10036)
Mouse IgG2a monoclonal anti-p17 (NIBSC CFAR; Cat#0342; clone 4C9)
Alexa Fluor 488-conjugated rabbit anti-FITC (Invitrogen; Car#A-11090)
Cy3-conjugated goat anti-mouse IgG2a (Jackson ImmunoResearch; Cat#115-165-206)
Alexa Fluor 647-conjugated anti!human IgG1 (H+L) antibody (Life Technologies; Cat#A-21445)
Anti-human IgG antibody coupled to 12 nm colloidal gold beads (Jackson ImmunoResearch;Cat#109-205-088).

Validation

The anti-HIV-1 Env antibodies specificity was validated in previous studies as they are unable to recognize non-infected cells (e.g. https://doi.org/10.1038/ncomms10844 ; https://doi.org/10.1128/JVI.02440-16)

Commercial antibodies were validated by the manufacturer:
PE-conjugated anti-CD4 antibody (clone VIT4; Miltenyi): https://www.miltenyibiotec.com/US-en/products/cd4-antibody-anti-human-vit4.html#gref

FITC-conjugated anti-Gag antibody (clone KC57; Beckman-Coulter): https://www.beckman.es/reagents/coulter-flow-cytometry/antibodies-and-kits/single-color-antibodies/hiv-1-core-antigen/6604667
Anti-alpha-Tubulin (clone AA13; Sigma-Aldrich): https://www.sigmaaldrich.com/ES/es/product/sigma/t8203
Polyclonal anti-p24 (NIH AIDS Reagent Program; Cat# ARP-4250): https://www.hivreagentprogram.org/Catalog/HRPPolyclonalAntiserum/ARP-4250.aspx
DyLight 680-conjugated Goat anti-Mouse IgG (Invitrogen; Cat#35518): https://www.thermofisher.com/antibody/product/Goat-anti-Mouse-IgG-H-L-Secondary-Antibody-Polyclonal/35518
DyLight 800-conjugated Goat anti-Rabbit IgG (Invitrogen; Cat#SA5-10036): https://www.thermofisher.com/antibody/product/Goat-anti-Rabbit-IgG-H-L-Cross-Adsorbed-Secondary-Antibody-Polyclonal/SA5-10036
Mouse IgG2a monoclonal anti-p17 (NIBSC CFAR; Cat#0342; clone 4C9): https://www.nibsc.org/science_and_research/idd/cfar/reagents_catalogue/product_details.aspx?ID=0342
Alexa Fluor 488-conjugated rabbit anti-FITC (Invitrogen; Car#A-11090): https://www.thermofisher.com/antibody/product/Fluorescein-Oregon-Green-Antibody-Polyclonal/A-11090
Cy3-conjugated goat anti-mouse IgG2a (Jackson ImmunoResearch; Cat#115-165-206): https://www.jacksonimmuno.com/catalog/products/115-165-206
Alexa Fluor 647-conjugated anti!human IgG1 (H+L) antibody (Life Technologies; Cat#A-21445): https://www.thermofisher.com/antibody/product/Goat-anti-Human-IgG-H-L-Cross-Adsorbed-Secondary-Antibody-Polyclonal/A-21445
Anti-human IgG antibody coupled to 12 nm colloidal gold beads (Jackson ImmunoResearch;Cat#109-205-088): https://www.jacksonimmuno.com/catalog/products/109-205-088

Eukaryotic cell lines

Policy information about cell lines

Cell line source(s)	293T cells (ATCC CRL- 3216) were obtained from ATCC. Freestyle 293-F were from ThermoFisher.
Authentication	Cell lines were not authenticated.
Mycoplasma contamination	All cells are negative for mycoplasma contamination. Tests were performed on a monthly basis.
Commonly misidentified lines (See ICLAC register)	None.

Human research participants

Policy information about studies involving human research participants

Population characteristics	16 HIV-1-infected individuals under antiretroviral therapy. Their demographic and clinical characteristics (age, gender, viral load, CD4 T cell count and ART duration) are presented in Supplementary Table 2.
Recruitment	Patients were recruited and sampled during their usual appointment with the physician that follows them for their HIV-1 infection. Any potential self-selection bias is unlikely to impact the results.
Ethics oversight	Each participant provided written consent to participate in the study, which was approved by the regional investigational review board (IRB; Comité de Protection des Personnes Ile-de-France VII, Paris, France) and performed according to the European guidelines and the Declaration of Helsinki.

Note that full information on the approval of the study protocol must also be provided in the manuscript.

Flow Cytometry

Plots

Confirm that:

- [x] The axis labels state the marker and fluorochrome used (e.g. CD4-FITC).
- [x] The axis scales are clearly visible. Include numbers along axes only for bottom left plot of group (a 'group' is an analysis of identical markers).
- [x] All plots are contour plots with outliers or pseudocolor plots.
- [x] A numerical value for number of cells or percentage (with statistics) is provided.

Methodology

Sample preparation	Cells were washed and incubated with a PE-conjugated anti-CD4 antibody (clone VIT4; Miltenyi; dilution 1:200) for 30 min at 4°C. Cells were fixed with 4% PFA for 10 minutes at room temperature. Cells were washed and stained with a FITC-conjugated anti-Gag antibody (clone KC57; Beckman-Coulter; diluted 1:500 in PBS/BSA 1%/Saponin 0.05%) for 30 min at room temperature. Cells were washed and resuspended in PBS. Data were acquired on an Attune NxT flow cytometer (Life Technologies) and analyzed using the FlowJo software (v10.7; BD).
Instrument	Attune NxT Acoustic Focusing Cytometer, blue/red/violet/yellow (catalog number : 15360667)

Software	AttuneNxT Software v3.2.1
Cell population abundance	At least 10,000 cells were acquired for each condition.
Gating strategy	All gates were set on CD4 T cells cultured without antibodies.

☒ Tick this box to confirm that a figure exemplifying the gating strategy is provided in the Supplementary Information.
